# Supplementary material for: Predictive Value of the Third Ventricle Width for Neurological Status in Multiple Sclerosis
Source: J Clin Med. 2022 May 18;11(10):2841. doi: 10.3390/jcm11102841 (PMC9145351; doi:10.3390/jcm11102841)
Supplement: Supplementary file 1 [file jcm-11-02841-s001.zip › jcm-1676936-supplementary.pdf]

Supplementary Data S1

The multiple regressions equations for each neuropsychological test with age, sex and education as predictors, calculated in the HPs group:

1) CVLT

   f (age, sex, edu) = 38.73727 – 0.22411 × age + 1.84064 × sex + 1.43405 × edu

   SD of the residuals = 8.971529

2) BVMT-R

   f (age, sex, edu) = 27.65302 – 0.18987 × age – 0.33783 × sex + 0.52458 × edu

   SD of the residuals = 5.463963

3) SDMT

   f (age, sex, edu) = 40.94645 – 0.35697 × age + 0.28770 × sex + 1.44756 × edu

   SD of the residuals = 10.58188
